# Pain Intensity Recognition Rates via Biopotential Feature Patterns with Support Vector Machines

**DOI:** 10.1371/journal.pone.0140330

**Published:** 2015-10-16

**Authors:** Sascha Gruss, Roi Treister, Philipp Werner, Harald C. Traue, Stephen Crawcour, Adriano Andrade, Steffen Walter

**Affiliations:** 1 University of Ulm, Medical Psychology, Department of Psychosomatic Medicine and Psychotherapy, Ulm, Germany; 2 Massachusetts General Hospital & Harvard Medical School, Department of Neurology, Nerve Injury Unit, Boston, Massachusetts, United States of America; 3 Otto-von-Guericke-Universität Magdeburg, Institute for Information Technology and Communications, Magdeburg, Germany; 4 Federal University of Uberlândia, Biomedical Engineering Laboratory (BioLab), Uberlândia, Brazil; 5 University of Technology Dresden, Department of Clinical Psychology and Psychotherapy, Dresden, Germany; Nanjing University of Aeronautic and Astronautics, CHINA

## Abstract

**Background:**

The clinically used methods of pain diagnosis do not allow for objective and robust measurement, and physicians must rely on the patient’s report on the pain sensation. Verbal scales, visual analog scales (VAS) or numeric rating scales (NRS) count among the most common tools, which are restricted to patients with normal mental abilities. There also exist instruments for pain assessment in people with verbal and / or cognitive impairments and instruments for pain assessment in people who are sedated and automated ventilated. However, all these diagnostic methods either have limited reliability and validity or are very time-consuming. In contrast, biopotentials can be automatically analyzed with machine learning algorithms to provide a surrogate measure of pain intensity.

**Methods:**

In this context, we created a database of biopotentials to advance an automated pain recognition system, determine its theoretical testing quality, and optimize its performance. Eighty-five participants were subjected to painful heat stimuli (baseline, pain threshold, two intermediate thresholds, and pain tolerance threshold) under controlled conditions and the signals of electromyography, skin conductance level, and electrocardiography were collected. A total of 159 features were extracted from the mathematical groupings of *amplitude*, *frequency*, *stationarity*, *entropy*, *linearity*, *variability*, and *similarity*.

**Results:**

We achieved classification rates of 90.94% for baseline vs. pain tolerance threshold and 79.29% for baseline vs. pain threshold. The most selected pain features stemmed from the *amplitude* and *similarity* group and were derived from facial electromyography.

**Conclusion:**

The machine learning measurement of pain in patients could provide valuable information for a clinical team and thus support the treatment assessment.

## Introduction

Quantifying pain is possible with the aid of the Visual Analog Scale or Numeric Rating Scale. However, these methods only work when the patient is sufficiently alert and cooperative, i.e., under conditions not always given in the medical field (e.g., post-surgery phases). There also exist instruments for pain assessment in people with verbal and / or cognitive impairments and instruments for pain assessment in people who are sedated and automated ventilated [1]. Overall, these methods are still in development or in need of validation. If conditions do not allow for a sufficiently valid measurement of pain, then cardiac stress in at-risk patients, under-perfusion of the operating field, or the development of chronic pain may follow in consequence. On the other hand, opiates may alleviate pain sensation, but can also lead to severe addiction, constipation etc. [2]. Hence, the measurement of biopotentials via the autonomic nervous system may be a solution that would permit an objective, reliable, and variable surrogate measurement of pain.

Some studies examined the correlation between a single biopotential and pain [3], [4], [5], [6], [7], [8], [9], [10]. In conventional pain diagnostics it is known that a measurement on a single parameter feature is insufficient for a valid diagnosis. Instead, a combination of multi-parameter features is required [1]. To the best of our knowledge, the study by Treister et al. [11] was the first to take a multi-parameter biopotential approach. Tonic heat was applied to elicit pain for a duration of 1 min, with intensities of “no pain,” “low pain,” “medium pain,” and “high pain.” While all of the features differed significantly in “no pain” and the other thresholds via Friedman Test, only the linear combination of parameters significantly differentiated between pain vs. no pain, as well as between all other pain categories. Bustan et al. also employed multimodal parameters to investigate the relationship between pain intensity, unpleasantness, and suffering [12]. High-intensity stimulation elicited higher skin conductance level compared with low-intensity stimulation under conditions of tonic noxious stimulation; heart rate was higher for short than for long stimulation, while corrugator electromyography showed of no significant effect regarding the response.

In our research we aim at the advancement of pain diagnosis and monitoring of pain states. For the purpose we developed an extensive multimodal dataset in which several levels of pain are induced. In a high density feature space a machine learning model (based on SVM) could be a solution. *“An SVM Model could be trained on one set of individuals*, *and used to accurately classify pain in different individuals”*, *p*. *2* [13].

In Walter et al. [14] preliminary results were presented. A total of 135 features were extracted from the mathematical groupings of *amplitude*, *frequency*, *stationarity*, *entropy*, *linearity*, and *variability* from the facial and trapezius electromyography, skin conductance level, and electrocardiography signals. The following features were statistically chosen as the most selective: 1. *electromyography_corrugator_amplitude_peak_to_peak*, 2. *electromyography_corrugator_entropy_ shannon*, and 3. *heart_rate_variability_slope_RR*. We received a classification rate (based on SVM) for the two class problem baseline vs. pain tolerance threshold of 77.05%. In Werner et al. [15] we received a classification rate for the two class problem baseline vs. pain tolerance threshold of 75.6% (without facial electromyography).

An extension of the feature space and the use of automated feature selection methods could improve classification rates, as compared to the study of Walter et al. [14].

**Fig 1:**
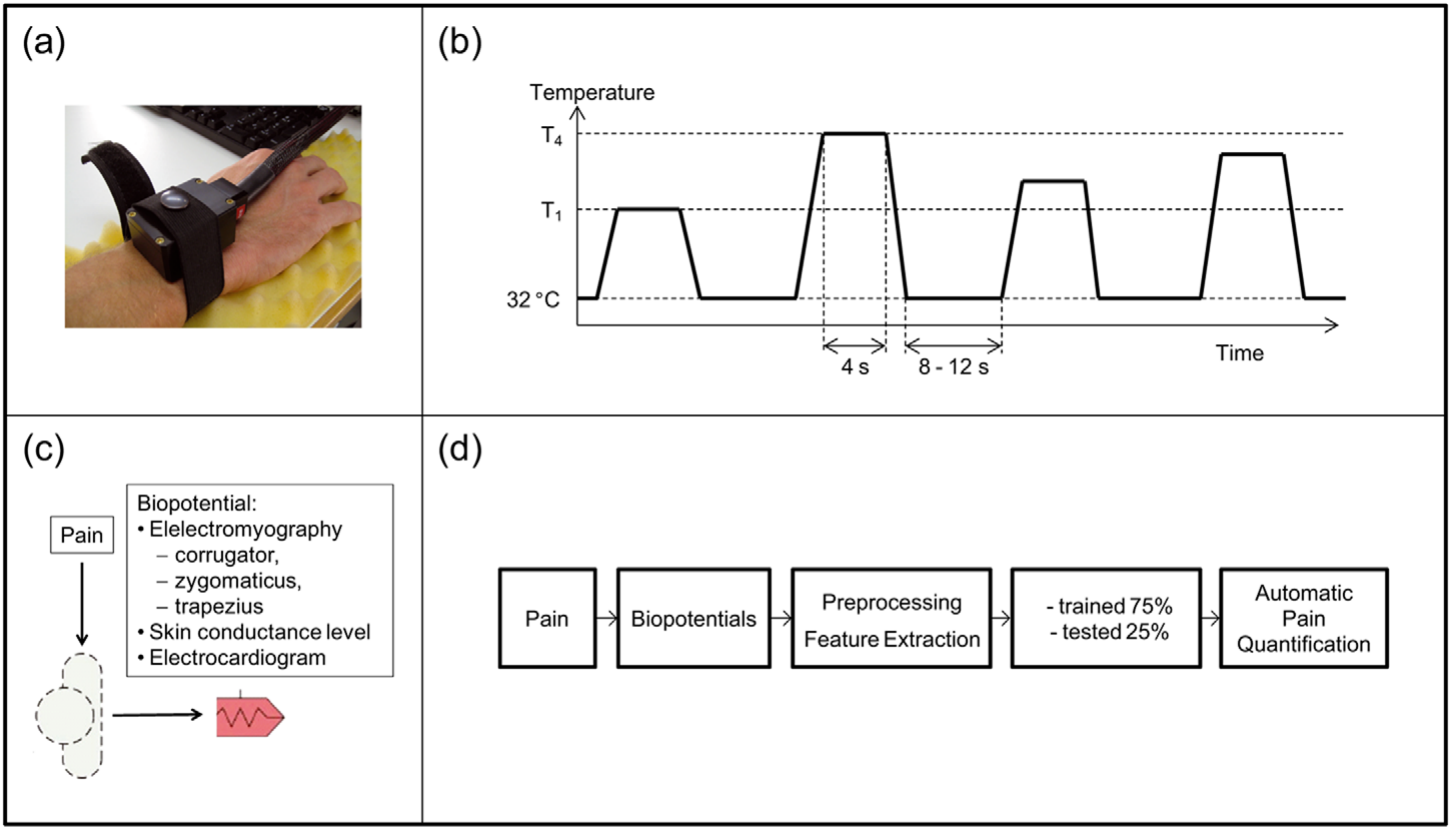
Experimental procedure, (1a) Thermode on the right arm, (1b) Heat signal with baseline, (1c) Labor setting, (1d) Study procedure.

The aim of the present study (see Fig 1d) was:

1. to select features (“pain pattern”) with a support vector machine learning design
2. to extract systematically a high-density feature space, for which we included signal similarity features (see Table 1, Equation: 37–42)—Similarity is mentioned in the signal processing literature as a powerful feature [16], [17]
3. to contribute to the highest recognition rate for pain quantification in a two and multi class problem.

**Table 1:** Feature information.

Number	Mathematical group	Feature name	Equation / Description
1	amplitude	peak	peak = max(signal); index(max(signal))
2	amplitude	p2p	p2p = max(signal)—min(signal)
3	amplitude	rms	rms = rms(signal)
4	amplitude	mlocmaxv	maxlocmaxv = mean(locmax(signal))
5	amplitude	minlocminv	minlocminv = mean(locmix(signal))
6	amplitude	mav	mav = mav(signal)
7	amplitude	mavfd	mavfd = mavfd(signal)
8	amplitude	mavfdn	mavfdn = mavfdn(signal)
9	amplitude	mavsd	mavsd = mavsd(signal)
10	amplitude	mavsdn	mavsdn = mavsdn(signal)
11	frequency	zc	Calculated by comparing each point of the signal with the next; if there is a crossing by zero then it is accounted.
12	frequency	fmode	This fast Fourier transformation equation is valid for this and the following frequency features: $X\left(k\right)=\Sigma_{j=1}^{N}x\left(j\right)\omega_{N}{}^{\left(j-1\right)\left(k-1\right)}$, where $\omega_{N}=e^{\left(-\frac{2\pi i}{N}\right)}$. To find the mode, find the maximum value of *X*.
13	frequency	bw	To obtain the bandwidth of a signal, find the first and the last frequencies where the spectral density values *X*(*kl*) and *X*(*kh*) are approximately 0.707*X(*kmax*), where *X*(*kmax*) is the maximum value of *X*. Finally, the bandwidth value is the subtraction of the frequency of *kh*(*fh*) by the frequency of *kl*(*fl*).
14	frequency	cf	The central frequency is simply the mean of the frequencies that delimit the bandwidth: $cf=\frac{fh-fl}{2}$.
15	frequency	fmean	$\frac{\sum_{k=1}^{NFFT}X\left(k\right).f\left(k\right)}{\sum X\left(k\right)}$
16	frequency	fmed	To obtain the median frequency, find the value of the frequency that bisects the area below the *X* waveform.
17	stationarity	median	$DS\;=\;\frac{1}{T}\int_{0}^{T}\left(1-\frac{H\;(\omega ,t)}{h\;(\omega )/T}\right)$ ^2^ *dt*, where *H*(*ω*, *t*) is the value of the spectrogram for frequency *ω* and time *t*, and *h*(*ω*) is the spectral density for frequency *ω*.
18	stationarity	freqpond	see description 17 above
19	stationarity	area	see description 17 above
20	stationarity	area_ponderada	see description 17 above
21	stationarity	me	Given the signal *x*, split it into *x*_*1*_, *x*_*2*_,… *x*_*n*_, where $n\;=\;\frac{T}{Ti}$, with *T* as the total time length of the signal, which is $\frac{N}{Fs}$, and *T*_*i*_ the time of each part *x*_*i*_. For each *x*_*i*_, compute the mean, then the standard deviation of the resultant mean vector.
22	stationarity	sd	Use the same split logic as in the previous feature. For each *x*_*i*_, compute the standard deviation, then the standard deviation of the resultant standard deviation vector.
23	entropy	aprox	For a temporal series with N samples {u(i): 1≤ *i* ≤*N*} given *m*, create vectors $X_{j}^{m}$ for each $X_{N-m+1}^{m}$ as $X_{j}^{m}$ = {u(i), u(i + 1),…, u(i + m − 1)}, i = 1,…, N − m +1, where *m* is the number of points to group together for the comparison. For each k ≤N-m+1 groups, do $C_{k}^{m}$(r) which is the number of times the groups had distance less than tolerance r. Then compute the value $\emptyset^{m}$ as $\;\emptyset^{m}(r)\;=\;\frac{\sum_{i\;=\;1}^{N-m+1}ln\;C_{i}^{m}(r)\;}{\left(N-m+1\right)}.$ The Approximated Entropy is: ApEn(m,r) = ${\text{lim}}_{N\to \infty }\left[\emptyset^{m}\left(r\right)-\;\emptyset^{m+1}\left(r\right)\right]$
24	entropy	fuzzy	$Saen(m,\;s,\;d)\;=\;ln\left[\frac{{Co}_{m}(s)}{{Co}_{m+1}(s)}\right]$, where *m* is the window size, *s* is the similarity standard and *d* is the signal. It is calculated in a very similar way to the Sample Entropy. The only similarity between the groups is computed by means of a Fuzzy membership function.
25	entropy	sample	$Saen(m,\;s,\;d)\;=\;ln\left[\frac{C_{m}(s)}{C_{m+1}(s)}\right]$, where *m* is the window size, *s* is the similarity standard and *d* is the signal. *C*_*m*_ is the regularity or frequency of similar windows in a given set of windows *d* with length *m*, obeying *s* tolerance.
26	entropy	shannon	$H\;=\;-\sum P_{k}\text{log}P_{k}$, where *P*_*k*_ is the probability of a value for each value present in a signal.
27	entropy	spectral	$S\;=\;\sum p_{k\;}\text{log}p_{k}$ / log (N), where *p*_*k*_ is the spectral density estimation of each *f*_*k*_ frequency.
28	linearity	pldf	$t\;\sqrt{R_{o\;(k)1,\ldots ,\;k-1}^{2}}$, where $R_{0(k)1,\ldots ,\;k-1}^{2}\;=\;\frac{\;{SQR}_{1,\ldots ,\;k-1}-{SQR}_{1,\ldots ,k}}{{SQR}_{1,\ldots ,\;k-1}}$
29	linearity	ldf	${FDD}_{k}\;=\;t\;\sqrt{R_{o\;(k)}^{2}}$, where $R_{0(k)}^{2}\;=\;\frac{\;{SQT}_{0}\;-SQR_{\left(k\right)}}{{SQT}_{0}}$
30	variability	var	$\sigma^{2}\;=\;\frac{\sum_{i\;=\;1}^{N}{\left(x_{i}-\overset{-}{x}\right)}^{2}}{N-1}$
31	variability	std	$S\;=\;\sqrt{\sigma^{2}}$
32	variability	range	R = MAX(U)-MIN(U)
33	variability	intrange	$SI\;=\;\frac{Q_{3}-Q_{1}}{2}$
34	variability	meanRR	meanRR = mean(hr_RR_vector)
35	variability	rmssd	$rmssd\;=\;\sqrt{\frac{1}{N-1}\left(\overset{N-1}{\underset{i-1}{\sum }}{\left(RR_{i}-RR_{i-1}\right)}^{2}\right)}$
36	variability	slopeRR	slopeRR = regression(x, hr_RR_vector)
37	similarity	cohe_f_median	$C(f)\;=\;\frac{S_{xy}(f)}{\sqrt{S_{xx}(f)S_{yy}(f)}}$
38	similarity	cohe_mean	see description 37 above
39	similarity	cohe_pond_mean	see description 37 above
40	similarity	cohe_area_pond	see description 37 above
41	similarity	corr	$P_{x,y}\;=\;\frac{E(XY)-E(X)-E(Y)}{\sqrt{E(X^{2})-E^{2}(X)}\sqrt{E(Y^{2})-E^{2}(Y)}}$
42	similarity	mutinfo	I(A,B) = H(A)+H(B)-H(A,B)

## Methods

### Subjects

A total of 90 subjects participated in the experiment, recruited from the following age groups [18]: 1. 18–35 years (*N* = 30 years; 15 men, 15 women), 2. 36–50 years (*N* = 30; 15 men, 15 women), and 3. 51–65 years (*N* = 30; 15 men, 15 women). Only 85 subjects were included in the final analysis because four subjects had to be excluded due to limited data quality with regard to the EMG. Recruitment was performed through notices posted at the university for the 18- to 35-year-old age group and through the press for the 36-50- and 51-65-year-old age groups. Only healthy subjects were recruited. The subjects received an expense allowance. The study was conducted in accordance with the ethical guidelines set out in the WMA Declaration of Helsinki (ethical committee approval was granted: 196/10-UBB/bal). The study was approved according the ethics committee of the University of Ulm (Helmholtzstraße 20, 89081 Ulm, Germany). All participants provided a written informed consent to participate in this study. An official written document of the ethics committee approved this consent procedure.

### Exclusion criteria

Prior to the experiment, the case history of each applicant was assessed in order to identify persons who met the exclusion criteria. Pre-existing neurological conditions, chronic pain, cardiovascular diseases, regular use of pain medication, and use of pain medication immediately before the experiment were applied as exclusion criteria.

### Individual calibration of pain and tolerance threshold

To induce heat pain [18], a Medoc Pathway thermal stimulator was employed. The ATS thermode was attached to the right forearm of the subject (see Fig 1a). Before data recording commenced, we determined each participant’s individual pain threshold and pain tolerance, i.e., the temperatures at which the participant’s perception changed from heat to pain and the level at which the pain became unacceptable. We used these temperature thresholds for the lowest and highest pain levels and added two additional intermediate levels to obtain a ranged set of four equally distributed temperatures (see Fig 1b). The instruction given to subjects for determining the pain threshold was as follows: “Please press the stop button immediately when you experience a burning, stinging, piercing or pulling sensation in addition to the feeling of heat.” In order to determine the tolerance threshold, the following instruction was given: “Please press the stop button immediately when you can no longer tolerate the heat, taking into account the burning, stinging, piercing or pulling sensation.”

### Experimental pain stimulation

In the experiment, each of the four temperature levels was applied 20 times in randomized order, resulting in a total of 80 stimuli. The baseline (no pain) was 32°C. For each stimulus, the temperature was maintained for 4 s. The pauses between the stimuli were randomized between 8–12 s (see Fig 1b). The subjects had the option to abort the experiment immediately by pressing an emergency stop button. After the experiment, we asked the subject to apply a cold pack to the spot of the heat stimulation for at least 5 minutes.

### Measurement of biopotentials

#### Biopotentials

A Nexus-32 amplifier (http://www.mindmedia.nl; accessed May 23, 2014) was used to record biopotential data (see Fig 1c) during the experiment. Biopotential and event data were recorded using Biotrace software. The following parameters were included in the classification process [18].

#### Electromyography (EMG)

Electrical muscle activity is also an indicator of general psychophysiological stimulation, in which increased muscle tone is associated with increasing activity of the sympathetic nervous system. A decrease in somatomotor activity reflects predominantly parasympathetic stimulation. We used two-channel EMGs for zygomaticus, corrugator, and trapezius muscles. In the area of affective computing, the activity of zygomaticus combined with happiness and the corrugator with negative affectivity [19]. The activity of the trapezius is an indication of a high stress level, which is also to be expected when pain is being experienced.

#### Skin conductance level (SCL)

To measure the skin conductance level, two electrodes connected to the sensor were positioned on the index and ring fingers. Because the sweat glands are innervated exclusively sympathetically (i.e., without the influence of the parasympathetic nervous system), electrodermal activity is considered a good indicator of the “inner tension" of a person. This phenomenon can be reproduced in particular by the observation of a rapid increase in skin conductance within 1–3 s due to a simple stress stimulus (e.g., deep breathing, emotional excitement, or mental activity).

#### Electrocardiogram (ECG)

We measured the average action potential of the heart on the skin using two electrodes, one on the upper right and one on the lower left of the body. Common features of the ECG signal are heart rate, interbeat interval, and heart rate variability (HRV). Heart rate variability refers to the oscillation of the interval between consecutive heartbeats and has been used as an indication of mental effort and stress in adults [20].

### Preprocessing

**Fig 2:**
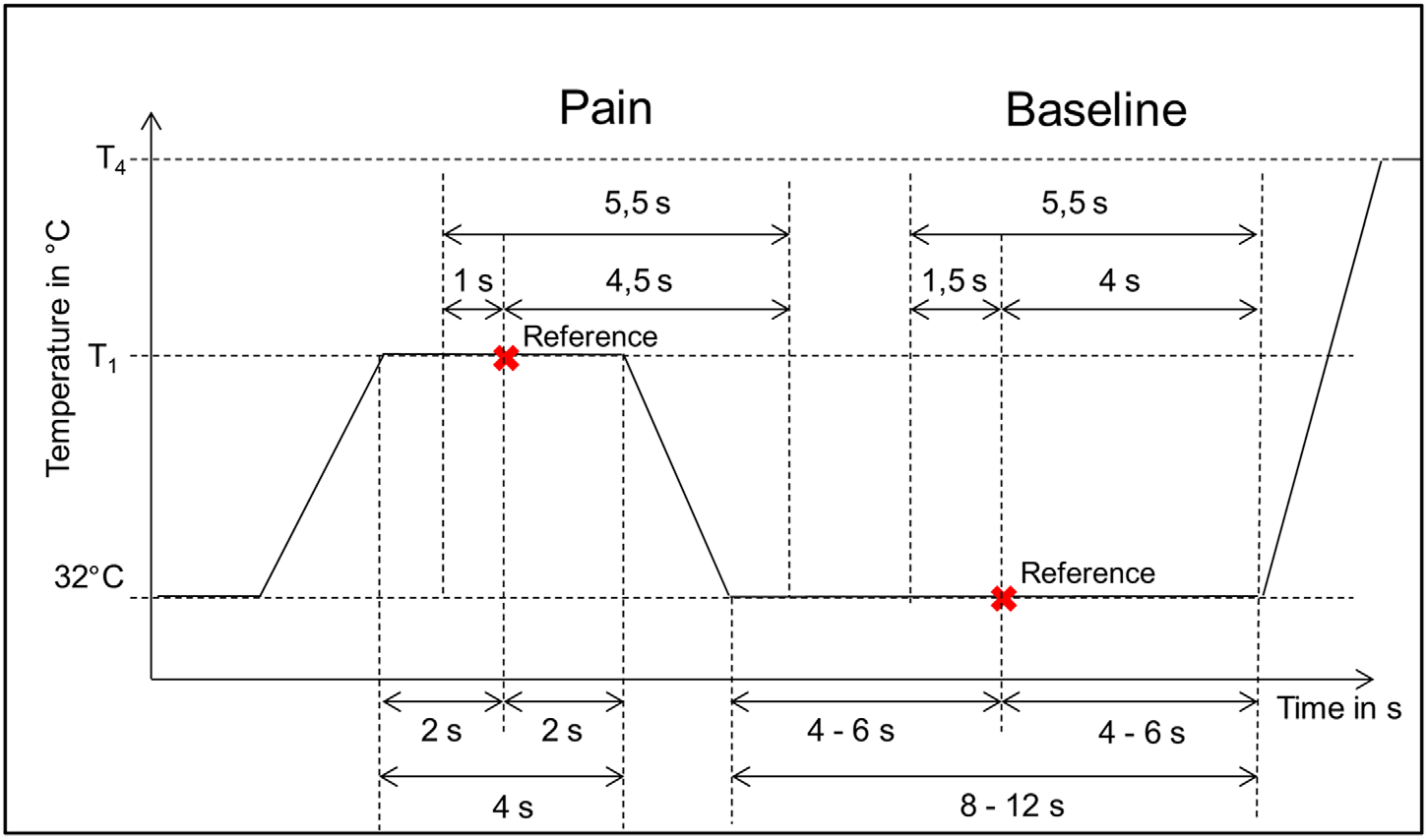
Pain quantification.

We performed the following biopotential preprocessing:

1. We visualized all biopotentials to check the intensity of the noise and activity with regard to pain stimulation.
2. We applied a Butterworth filter to the EMG (20–250 Hz) and ECG (0.1–250 Hz) signals.
3. For the EMG, we also applied an additional filter using the Empirical Mode Decomposition technique developed by [21].
4. We quantified the pain level caused by the heat applied using four pain thresholds during the “pain window” (5.5 s) and with regard to the baseline during the “non-pain window” (see Fig 2).
5. We detected bursts of EMG activity using the Hilbert Spectrum [22].

### Feature extraction

**Fig 3:**
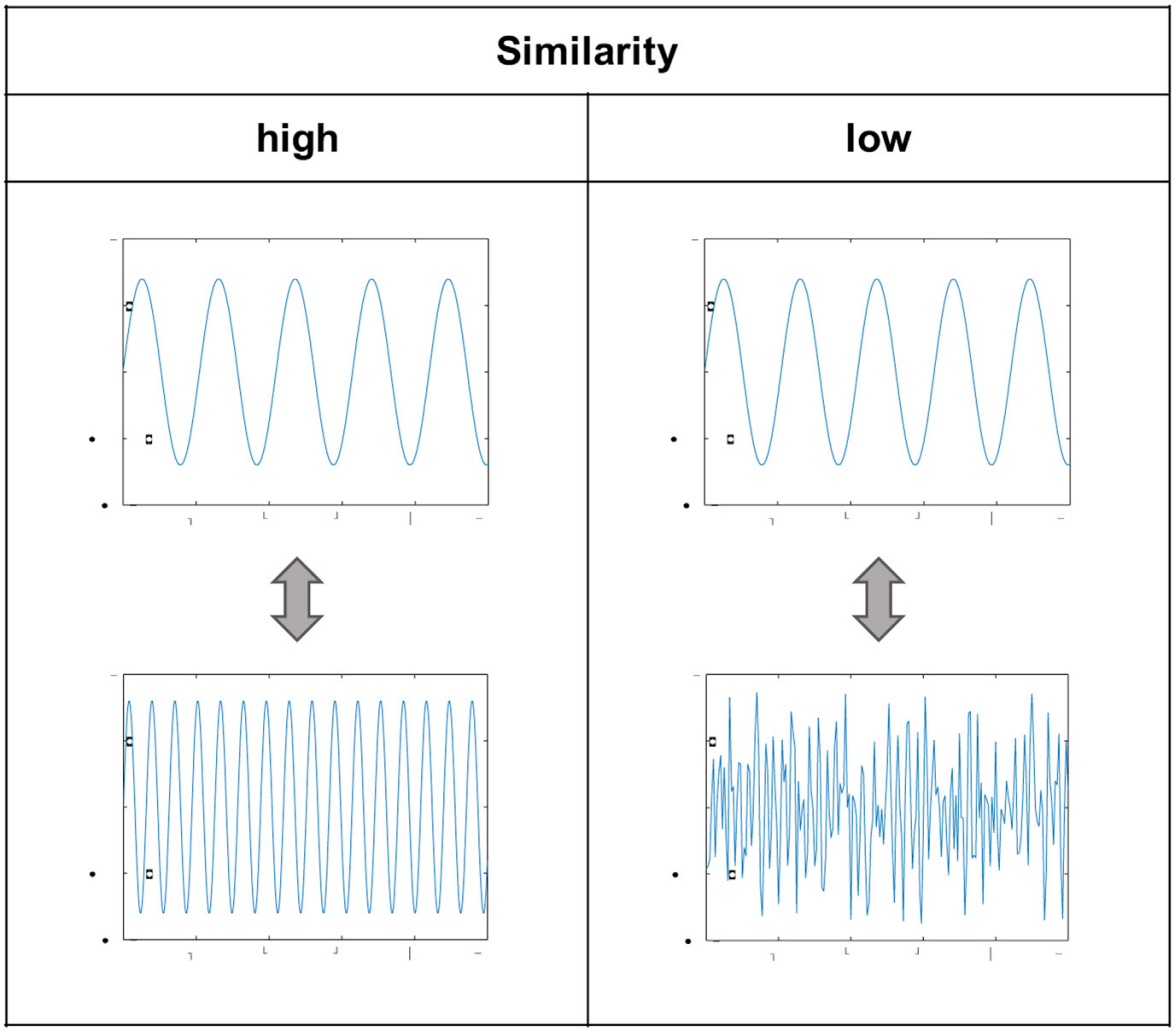
Similarity feature.

We systematically extracted features [23], [24], [25], [26] from the mathematical groups of 1. *amplitude* (∑ = 40), 2. *frequency* (∑ = 24), 3. *stationarity* (∑ = 24), 4. *entropy* (∑ = 20), 5. *linearity* (∑ = 8), 6. *variability* (∑ = 19) and 7. *similarity* (Fig 3) (∑ = 24) (in total: ∑ = 159). Table 1 provides a detailed information overview of all features. The similarity features of a sample are calculated with regard to the associated mean baseline signal of the person. All features were normalized (z transformed) per person. The dataset of the study, including the raw and preprocessed signals, as well as the extracted features, is available at: https://www-e.uni-magdeburg.de/biovid/.

### Machine learning with Support Vector Machine (SVM)

Machine learning systems are systems that learn from known data and attempt to recognize characteristic patterns. After a 'learning phase' (also referred to 'training phase'), they return a model that can be used to map (i.e., classify) unknown input data into a category [27]. For these classification tasks, there are several machine learners (classifiers), all of which work using different decision algorithms, such as Neural Networks, Decision Trees, K-Nearest Neighbor, and SVM.

**Fig 4:**
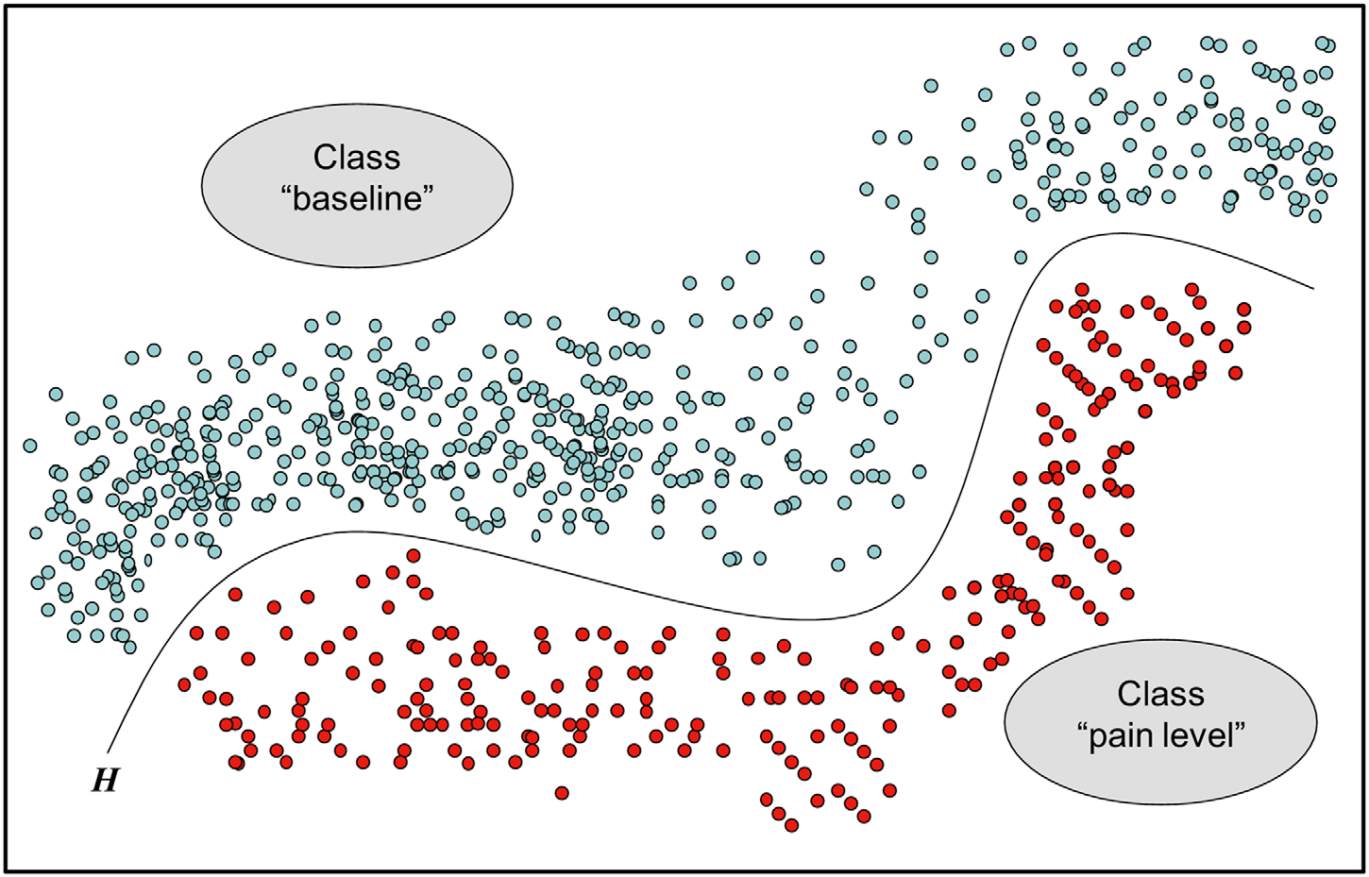
Support Vector Machine hyperplane (*H*).

For the classification of different pain intensities, we considered using Neural Networks and k-Nearest Neighbor, but finally we chose SVMs (see Fig 4), as these have proven to be highly effective in other studies of affective computing [28] and are capable of maintaining sufficient flexibility with regard to their internal main parameter optimization [29].

The goal of an SVM is to develop a predictive model based on the given training samples (*x*_*i*_, *y*_*i*_) with *x*_*i*_ being a feature vector and *y*_*i*_ its associated class label. This model can subsequently be applied to an unlabeled test dataset to assign a particular class to each sample. With the aid of the feature vectors *x*_*i*_, the SVM [30] searches for an optimal hyperplane with maximum margin in the feature space that separates the feature vectors of one class from feature vectors of the other. The hyperplane thus serves as the decision function. If the linear separation is not possible in the original feature space, all training vectors can be transformed to a higher dimensional space until the SVM finds a dividing hyperplane. This is done by means of a kernel function. In the present case we used a radial basis function kernel (RBF kernel), because it is able to handle non-linear dependencies between class labels and input attributes. Furthermore, the RBF-kernel has the advantage that the complexity of the model is limited to only two main parameters (*C*, *γ*). *C* controls the cost of misclassification of the training vectors [31], while *γ* controls the radius of influence of the support vectors [32]. In order to obtain optimal SVM parameters, a systematic grid search was performed for *C* and *γ* on 75% of data with a resulting 3-fold-cross-validation. When choosing the parameter values, we followed the procedure by Hsu et al. [29], who recommend exponentially growing sequences for *C* and *γ*: *C* = 2^−5^, 2^−3^,…, 2^15^ and *γ* = 2^−15^, 2^−13^,…, 2^3^. After testing of all combinations, the pair with the highest accuracy was finally selected as the optimal parameter set.

The performance of a classifier in general in a given learning task is measured by its classification rate (accuracy). Simply put, a set of feature vectors with known class labels is divided into two randomized mixed subsets. One subset is used for training the model and the other one is then applied for testing purposes. By putting the remaining feature vectors into the model, comparing their class labels with the classifier’s predictions, and finally counting the correctly predicted testing vectors, one receives the classification rate (accuracy) defined as

$$\frac{\text{number of correctly predicted testing vectors}}{\text{total number of testing vectors}}.$$ (1)

We also calculated sensitivity and specificity, which give further information about the performance of the classification task. Both statistical measures are derived from the confusion matrix of the task. Put the case we have class “positive” and class “negative” with corresponding testing vectors for “positive” and “negative”. Then sensitivity is defined as

$$\frac{\text{number of correctly predicted testing vectors for "positive"}}{\text{total number of testing vectors for class "positive"}}$$ (2)

and specificity as

$$\frac{\text{number of correctly predicted testing vectors for class "negative"}}{\text{total number of testing vectors for class "negative"}}.$$ (3)

### Feature selection

Automatic pattern selection methods are used to further optimize recognition rates. Feature selection is a “method for selecting a subset of features providing optimal classification accuracy of the classification model” [33]. This is accomplished by means of a variety of feature selection (pattern configuration) methods, in combination with a classification procedure.

Before we conducted an automatic feature selection algorithm, we performed a manual pre-selection of the extracted 159 features based on statistical analyses and validation checks. As a first step, we eliminated features (feature groups) containing either a zero or a static number for all conditions. Such values may be attributed to a compromised signal or to a special feature extraction algorithm that appeared as uninterpretable on a particular bio signal. Second, we deleted all features which correlated positively or negatively with other features with at least 0.95 respectively—0.95. By doing so, we attempted to prevent classification of noise and redundant information.

Finally, we applied a feature selection algorithm combined with pain classification tasks to the remaining features. For this step, we chose the forward selection as it has shorter run-times (in most of the cases) as the backward elimination and brute force selection. The forward selection algorithm starts with an empty feature set and adds a new feature in each round. At each round, every new feature is tested for inclusion in the set by calculating a classification accuracy. The feature with the most increased accuracy is then added to the set before the beginning of the next round. The algorithm runs until there is no increase anymore [34].

**Fig 5:**
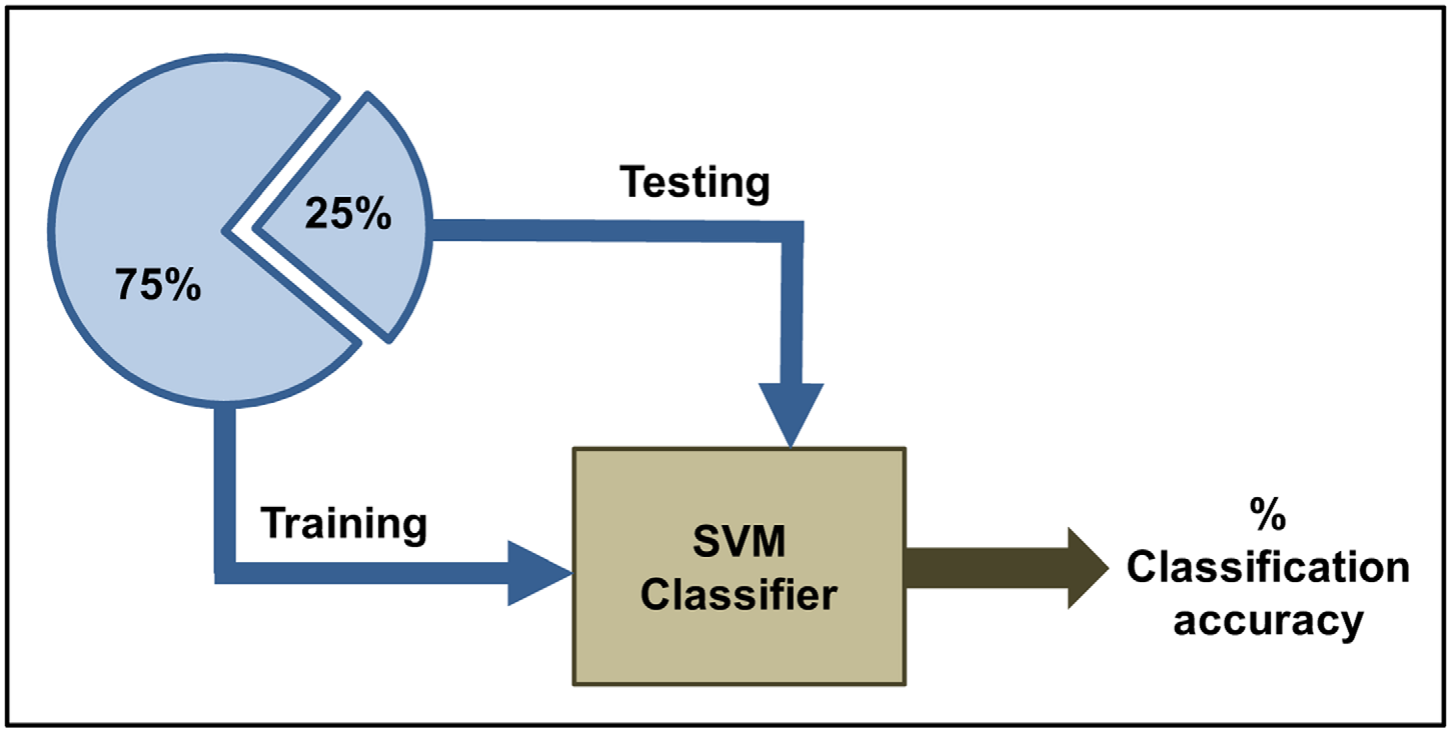
Support Vector Machine learning architecture.

We conducted the Support Vector Machine classification tasks in conjunction with the previously mentioned feature selection method on varying data sets and different biopotentials. SVMs were trained on 75% of the data (total number of training samples = 6375) tested on the remaining 25% of data (total number of test samples = 2125) (see Fig 5). The number of hereby-obtained optimal features ranges from 5 to 22 (depending on learning task).

### Validity of classification rates

In many papers that address classification tasks like emotion or pain detection, machine learning classification results are presented without a measure of the validity—like in conventional statistic the p-level or effect size. However, we think this should be included in future analyses. In our case, we chose *Cramér’s V* [35] as an appropriate measure as it measures the strength of association between nominal variables. The value of *Cramér’s V* is ranging from 0 to +1 and is interpreted as follows: *V* < 0.1: *negligible* association, 0.1 < *V* < 0.2: *weak* association, 0.2 < *V* < 0.4: *moderate* association, 0.4 < *V* < 0.6: *relatively strong* association, 0.6 < *V* < 0.8: *strong* association, and 0.8 < *V* < = 1: *very strong* association [36].

## Results

**Fig 6:**
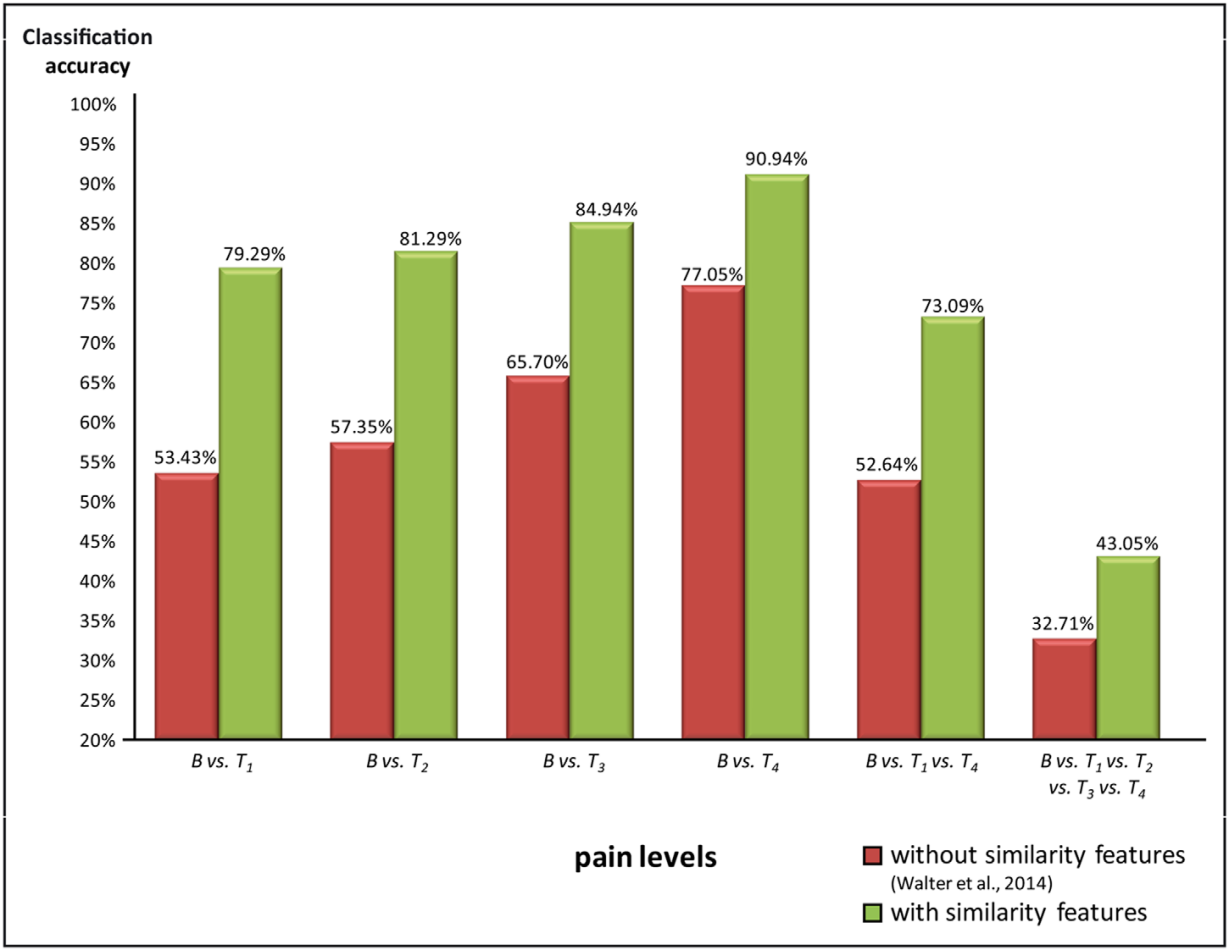
Comparison between accuracy via support vector machine of study Walter et al. [14], without similarity signal feature [red] vs. support vector machine with similarity feature and automated selected feature [green].

The classification results are summarized in Fig 6. Significance was tested against chance level. The classification results for a two class problem are between 79.29% - 90.94%, the *Cramer’s V* results and statistical measures sensitivity and specificity for *B* vs. *T*_*1*_ are *V* = 0.59 (sensitivity = 76.00%, specificity = 82.59%), *B vs*. *T*_*2*_ are *V* = .63 (sensitivity: 80.00%, specificity: 82.59%), *B vs*. *T*_*3*_ are 84.94% *V* = .7 (sensitivity = 84.71%, specificity = 85.18%) and *B vs*. *T*_*4*_ are 90.94% *V* = 0.82 (sensitivity = 92.24%, specificity = 89.65%).

The *Cramer’s V* results and statistical measures sensitivity and specificity for the three and five class problem are between 43.05% - 73.09%, the classification results for *B vs*. *T*_*1*_ *vs*. *T*_*4*_ are *V* = .62 (*B vs*. *others*: sensitivity = 78.64%, specificity = 70.18%; *T*_*1*_ *vs*. *others*: sensitivity = 69.96%, specificity = 74.69%; *T*_*4*_ *vs*. *others*: sensitivity = 70.71%, specificity = 74.34%) and for *B vs*. *T*_*1*_ *vs*. *T*_*2*_ *vs*. *T*_*3*_ *vs*. *T*_*4*_ are *V* = .38 (*B vs*. *others*: sensitivity = 76.32%, specificity = 34.50%; *T*_*1*_ *vs*. *others*: sensitivity = 34.89%, specificity = 45.25%; *T*_*2*_ *vs*. *others*: sensitivity = 15.95%, specificity = 49.74%; *T*_*3*_ *vs*. *others*: sensitivity = 28.18%, specificity = 46.52%; *T*_*4*_ *vs*. *others*: sensitivity = 58.71%, specificity = 39.22%). Table 2 points out the most common features in a ranked order.

**Table 2:** Top ten importance ranking of selected features.

rank	feature name
1	*Zygomaticus_Similarity_Correlation*
2	*Zygomaticus_Stationarity_StandardDeviationOfMeanVector*
3	*Corrugator_Amplitude_Peak*
4	*Corrugator_Similarity_Correlation*
5	*Corrugator_Similarity_MutualInformation*
6	*Trapezius_Similarity_Correlation*
7	*Zygomaticus_Amplitude_RootMeanSquare*
8	*Zygomaticus_Linearity_LagDependenceValues*
9	*Zygomaticus_Variability_Variance*
10	*Trapezius_Similarity_MutualInformation*

## Discussion and Conclusions

Our goal was to find significant classification results with high accuracies and an automated selected feature pattern of biopotentials that represents 'pain' and 'no-pain', respectively. We extracted a highly complex and structured mathematical feature space. We could show that (1) similarity features and an (2) automatic feature selection outperform the accuracy results from [14]. We found recognition rates with high accuracies based on a configuration of selected features. Especially the recognition rate for *B vs*. *T*_*4*_ (pain tolerance) showed a relatively high quality with regard to *Cramer’s V*. To the best of our knowledge, this is the first study in the area of automated pain recognition to employ biopotential data with accuracy over 90%. Highly relevant are features based on *similarity* derived from zygomaticus and corrugator. Similarly to other studies of automated pain recognition via video recording, we pointed out that facial expressions are highly relevant regarding the pain intensity. The importance of features derived from *similarity* thus needs to be tested systematically.

### Study weaknesses

We added two additional intermediate levels to obtain a ranged set of four equally distributed temperatures without a nonlinear correction.

### Outlook

Our intention is to optimize our classification algorithm. In reference to that, we are currently planning future experimental procedures.

1. **Generalizability**: We will test and improve the generalizability with a complex pain model (phasic and tonic, heat and pressure).
2. **Response Specificity**: The specificity of the recognition system will be evaluated with the task to distinguish pain from psychosocial stress.
3. **Assessment modalities**: In addition to psychobiological and facial parameters, we will assess paralinguistic properties, skin temperature, body movement, and other modalities for pain recognition.
4. **Reliability** of pain recognition will be tested by repeating the experiment after (at least) one week.
5. All algorithms will be adapted for **online processing** to advance towards a pain monitoring system.

Further, the classification algorithm requires testing and optimization within a clinical environment. Finally, the goal of the project is the advancement of pain diagnosis and monitoring of pain states. With the use of multimodal sensor technology and highly effective data classification systems, reliable and valid automated pain recognition will be possible. The surrogate measurement of pain with machine learning algorithms will provide valuable information with high temporal resolution for a clinical team, which may help to objectively assess the evolution of treatments (e.g., effect of drugs for pain reduction, information of surgical indication, the quality of care provided to patients).
